# The association between small airway dysfunction and aging: a cross-sectional analysis from the ECOPD cohort

**DOI:** 10.1186/s12931-022-02148-w

**Published:** 2022-09-04

**Authors:** Cuiqiong Dai, Fan Wu, Zihui Wang, Jieqi Peng, Huajing Yang, Youlan Zheng, Lifei Lu, Ningning Zhao, Zhishan Deng, Shan Xiao, Xiang Wen, Jianwu Xu, Peiyu Huang, Kunning Zhou, Xiaohui Wu, Yumin Zhou, Pixin Ran

**Affiliations:** 1 State Key Laboratory of Respiratory Disease, National Clinical Research Center for Respiratory Disease, Guangzhou Institute of Respiratory Health, the First Affiliated Hospital of Guangzhou Medical University, Guangzhou Medical University, No. 195 Dongfeng Xi Road, Guangzhou, 510000 Guangdong China; 2 Guangzhou Laboratory, Bio-Island, Guangzhou, Guangdong People’s Republic of China

**Keywords:** Small airway dysfunction, Aging, Computed tomography, Impulse oscillometry, Spirometry

## Abstract

**Background:**

Aging has been evidenced to bring about some structural and functional lung changes, especially in COPD. However, whether aging affects SAD, a possible precursor of COPD, has not been well characterized.

**Objective:**

We aimed to comprehensively assess the relationship between aging and SAD from computed tomography, impulse oscillometry, and spirometry perspectives in Chinese.

**Methods:**

We included 1859 participants from ECOPD, and used a linear-by-linear association test for evaluating the prevalence of SAD across various age subgroups, and multivariate regression models for determining the impact of age on the risk and severity of SAD. We then repeated the analyses in these subjects stratified by airflow limitation.

**Results:**

The prevalence of SAD increases over aging regardless of definitional methods. After adjustment for other confounding factors, per 10-yrs increase in age was significantly associated with the risk of CT-defined SAD (OR 2.57, 95% CI 2.13 to 3.10) and the increase in the severity of air trapping (β 2.09, 95% CI − 0.06 to 4.25 for LAA_-856_), airway reactance (β − 0.02, 95% CI − 0.04 to − 0.01 for X5; β 0.30, 95% CI 0.13 to 0.47 for AX; β 1.75, 95% CI 0.85 to 2.66 for Fres), as well as the decrease in expiratory flow rates (β − 3.95, 95% CI − 6.19 to − 1.71 for MMEF%predicted; β − 5.42, 95% CI − 7.88 to − 2.95 for FEF_50_%predicted) for SAD. All these associations were generally maintained in SAD defined by IOS or spirometry. After stratification of airflow limitation, we further found that the effect of age on LAA_-856_ was the most significant among almost all subgroups.

**Conclusions:**

Aging is significantly associated with the prevalence, increased risk, as well as worse severity of SAD. CT may be a more optimal measure to assess aging-related SAD. The molecular mechanisms for the role of aging in SAD need to be explored in the future.

*Trial* *registration* Chinese Clinical Trial Registry ChiCTR1900024643. Registered on 19 July 2019

**Supplementary Information:**

The online version contains supplementary material available at 10.1186/s12931-022-02148-w.

## Introduction

As we know, advancing age brings about some structural and functional lung changes. For example, in healthy old-age persons, enlarged alveoli, increased bronchial thickening, reduced surface area for gas change, and loss of alveolar attachment have been described as “senile emphysema” [1]. It is also evidenced that lung aging involved increased air trapping [2], increased residual volume, decreased vital capacity [3], and decreased expiratory flow rates [4]. Similarly, these physiological abnormalities also appear in Chronic Obstructive Lung Disease (COPD) [5], which has been widely recognized as an age-related disease.

Small airways are the peripheral airways with luminal diameters less than 2 mm [6]. Small airway dysfunction (SAD) is characterized by thickened small airway walls, reduced cross-sectional area of terminal bronchioles [7], decreased vital capacity, and increased residual volume [8]. Some pathological findings emphasized that substantial loss of small airways preceded the pathological evidence of emphysema and COPD [7]. SAD is also increasingly seen as a precursor for the development of COPD. This allowed us to raise a scientific question of whether SAD is also an abnormality associated with aging like COPD. In view of this, a letter to European Expiratory Journal’s editors showed that increasing age was associated with elevated levels of image abnormality including PRM^fSAD^ and PRM^Emph^ [9]. Martinez et al. found that in American subjects without airflow obstruction, aging is associated with functional small airway abnormality defined by computed tomography (CT), regardless of respiratory symptoms [10]. It was also reported in a study by Lee et al. that the prevalence of CT-defined air trapping increased with age in merely 82 asymptomatic Koreans [2]. However, these preliminary explorations only focused on Americans or Koreans merely from the CT perspective. The comprehensive associations between aging and SAD from the image, impulse oscillometry (IOS), and spirometry in Chinese remain not characterized.

There is no gold standard for SAD diagnosis for the difficulties in detecting small airway abnormalities. The current recommended and feasible criteria for measuring and defining SAD were mainly CT, IOS, and spirometry [11]. Thus, we comprehensively assessed the impact of aging on SAD from all these different measures. Identifying age-related changes in SAD from various perspectives will provide us with a better understanding of SAD and have direct implications for defining early lung diseases.

We had three main objectives in our study. First, we aimed to comprehensively evaluate the associations of aging with prevalence and the risk of SAD defined by CT, IOS, and spirometry methods. Second, we assessed the impact of aging on the severity of SAD indicated by a variety of SAD markers. Third, we compared what kind of markers is the most sensitive measure to disclose age-associated alterations in SAD and thus determined the possible optimum diagnostic method.

## Materials and methods

### Study participants

The data used in our study were from a cross-sectional analysis of the Early Chronic Obstructive Pulmonary Disease (ECOPD) study (Trial registration: Chinese Clinical Trial Registry ChiCTR1900024643). ECOPD was a prospective observational cohort study. The first participant was enrolled in July 2019. Baseline enrollment ended in August 2021. Details regarding rationale, design, and inclusion as well as exclusion criteria have been illustrated elsewhere previously [12]. Briefly, approximately 2000 participants aged 40–80 years were recruited voluntarily in Guangdong, China. These participants consisted of approximately 1000 subjects with COPD and the others without COPD. COPD was defined as post-bronchodilator forced expiratory volume in the first second (FEV_1_)/forced vital capacity (FVC) < 0.7 [13]. All the participants completed the tests including standard respiratory epidemiological questionnaires, spirometry, high-resolution CT, IOS, and so on at baseline. And they will be followed up every year. This study focused on subjects with eligible questionnaires, CT, IOS, and spirometry (N = 1859). We assessed the association of aging with SAD from all these different measures. Furthermore, given the substantial effects of airflow limitation on spirometry-defined SAD and the characteristics of enrollment in ECOPD, we repeated all the analyses in the same 1859 subjects stratified by airflow limitation, which was defined as FEV_1_/FVC < 0.7. The ECOPD study was approved by the Ethics Committee of the First Affiliated Hospital of Guangzhou Medical University (No.2018-53), and written informed consent was attained from all the participants.

### Questionnaire

The standard respiratory epidemiological questionnaire, which has been modified from an international BOLD study [14] and had been used in previous COPD studies in China [15, 16], was conducted by trained investigators in our study. We extracted information regarding demography and risk factors for COPD. Besides, we also calculated the modified Medical Research Council and COPD Assessment Test [17]. We collapsed known annual household income into three levels (low: < 10,000 RMB; medium: 10,000–100,000 RMB; high: > 100,000 RMB). Education level was categorized into primary school or below, middle or high school, and college or above. Except for these two parameters, definitions of the other variables were the same as those described before [12].

### CT image analysis

High-resolution CT was performed by our professional researchers. We used a multidetector-row CT scanner (Siemens Definition AS Plus 128-slicers and United-imaging uCT 760 128-slicers) to get the images. Complete details of the CT protocol and quality control have been outlined previously [12]. Quantitative analysis of CT images was performed using Chest Imaging Platform (https://www.chestimagingplatform.org) on the semi-automated 3D Slicer 4.11 software (https://www.slicer.org) [18]. Emphysema was quantified using the percentage of low attenuation units less than -950 HU at full inspiration (LAA_-950_), and gas trapping using the percentage of low-attenuation units less than -856 HU at full expiration (LAA_-856_) [19]. Besides, we also recorded the parameters such as residual volume (RV) and total lung capacity (TLC). The severity of CT-defined SAD was indicated by parameters including LAA_-950,_ LAA_-856,_ RV, and TLC. Someone with LAA_-856_ > 20% was recognized as SAD in this study [20].

### Impulse oscillometry

We performed IOS (MasterScreen IOS, Hochberg, Germany) in accordance with European Respiratory Society guidelines [21]. IOS was conducted before premedication and spirometry for the influence of forced expiration itself on airway tone [22]. All the data were reviewed by experts to achieve high quality. We recorded the following IOS parameters: resistance at 5 Hz (R5), resistance at 20 Hz (R20), the difference between R5 and R20 (R5–R20), reactance at 5 Hz (X5), reactance area (AX), resonant frequency (Fres). Of these, R5–R20 reflects the resistance of small or peripheral airways. Its value > 0.07 Ka/L/s indicated SAD [23]. The severity of IOS-defined SAD was indicated by all these recorded parameters about airway resistance and reactance.

### Spirometry

We conducted spirometry using conventional spirometers (Carefusion MasterScreen Pneumo, Germany). All the participants underwent a lung function test with a bronchodilator administration (Salbutamol Sulfate Aerosol, 400 μg, 20 min later). The operational maneuvers and quality control standards were carried out according to the American Thoracic Society and Europe Respiratory Society [24, 25]. The maximal mid-expiratory flow of percent predicted (MMEF%predicted), forced expiratory flow at 50 and 75 of forced vital capacity of percent predicted (FEF_50_%predicted and FEF_75_%predicted) were all taken from the best curves with the largest sum of FEV_1_ and FVC. SAD was defined if post-bronchodilator MMEF %predicted, FEF_50_%predicted or FEF_75_%predicted (any two of the three) are < 65% [26]. The severity of spirometry-defined SAD was indicated by these three parameters for mid-to-end expiratory flow rates.

### Statistical analyses

The details can be found in the statistical analyses section from supplemental materials.

## Results

### Characteristics of participants

**Fig. 1:**
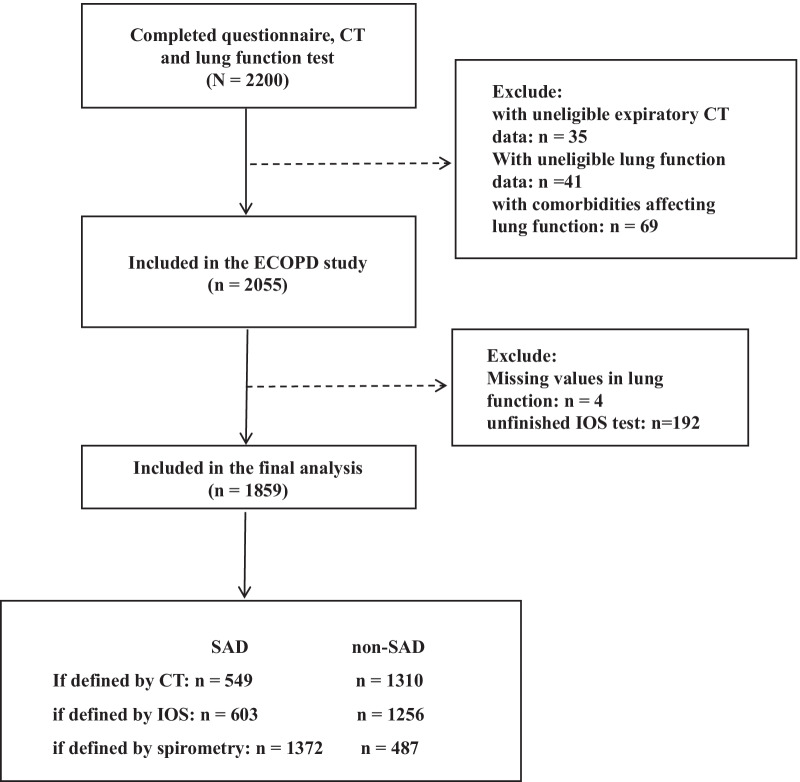
Flow chart for included subjects selection and stratified by definitional methods. *SAD* small airway dysfunction, *CT* computed tomography, *IOS* impulse oscillometry

By August 2021, 2200 participants have completed the questionnaire, CT, and spirometry examinations. After the eligibility assessment, 341 subjects were excluded. The details of exclusion were listed in Fig. 1. The remaining 1859 participants were included in the final analysis and assessed from different perspectives (CT, IOS, and spirometry). Then, we repeated all the analyses in these subjects after stratifying by airflow limitation. The details of stratification were listed in Additional file 1: Fig. S1.

Table 1 shows that the mean age in our cohort was 61 years old and males comprised 74% of the total subjects. After stratification, compared with the nonobstructive subgroup, individuals in the airflow limitation subgroup were more likely to be older, male, underweight; have more cigarette or occupational exposure, greater respiratory symptoms without colds, more comorbidities, more smokers living at home, and more parental history of respiratory diseases; have a greater proportion of people at low or medium educational levels, low or medium annual household income and exacerbation; have worse health status (higher mMRC and CAT scores). Besides, the obstructive subgroup had worse lung function, greater airway resistance and reactance, and greater abnormalities on CT images than the nonobstructive subgroup.

**Table 1:** Characteristics of total subjects and stratified by airflow limitation

Characteristics	Total (n = 1859)	Without airflow limitation (n = 1027)	Airflow limitation (n = 832)	P value
Age, y	61.3 ± 8.2	58.5 ± 7.9	64.7 ± 7.1	< 0.001
Male sex, %	1375 (74.0)	613 (59.7)	762 (91.6)	< 0.001
BMI, kg/m^2^	22.7 ± 3.2	23.3 ± 3.2	22.1 ± 3.2	< 0.001
Educational level, %				< 0.001
Primary school or less	882 (47.4)	501 (48.8)	381 (45.7)	
Middle or high school	927 (49.9)	487 (47.4)	440 (52.9)	
College or higher	50 (2.7)	39 (3.8)	11 (1.3)	
Annual household income, %				< 0.001
Low	370 (19.9)	174 (16.9)	196 (23.6)	
Medium	1286 (69.2)	747 (72.7)	539 (64.9)	
High	130 (7.0)	87 (8.5)	43 (5.2)	
Unknown	73 (3.9)	19 (1.9)	54 (6.5)	
Smoking status, %				< 0.001
Never smoker	649 (34.9)	538 (52.4)	111 (13.4)	
Former smoker	385 (20.7)	133 (13.0)	252 (30.2)	
Current smoker	825 (44.4)	356 (34.7)	469 (56.4)	
Smoking index, pack-years	27.3 ± 31.5	19.6 ± 29.0	36.9 ± 31.8	< 0.001
Smokers living at home, %	732 (39.4)	430 (41.9)	302 (36.3)	0.324
Parental history of respiratory diseases, %	245 (13.2)	91 (8.9)	154 (18.5)	< 0.001
Occupation exposure > 6 months, %	382 (20.6)	168 (16.4)	214 (25.8)	< 0.001
Indoor exposure to biomass for cooking or heating, %	662 (35.6)	351 (34.2)	311 (37.4)	0.348
History of comorbidities, %				
Asthma	27 (1.5)	5 (0.5)	22 (2.6)	0.007
Tuberculosis	47 (2.5)	18 (1.8)	29 (3.5)	0.034
Chronic bronchitis	93 (5.0)	31 (3.0)	62 (7.5)	0.007
History of prior year exacerbation, %	184 (9.9)	63 (6.1)	121 (14.6)	< 0.001
Respiratory symptoms without colds, %				
Cough	520 (27.9)	179 (17.4)	341 (41.0)	< 0.001
Sputum	657 (35.3)	248 (24.1)	409 (49.2)	< 0.001
Wheeze	209 (11.2)	58 (5.6)	151 (18.1)	< 0.001
mMRC	0.37 ± 0.62	0.25 ± 0.51	0.52 ± 0.70	< 0.001
CAT	4.28 ± 6.80	3.64 ± 7.76	5.06 ± 5.28	< 0.001
Spirometry parameters after bronchodilator use				
FEV_1_/FVC,	69.8 ± 12.9	79.0 ± 5.6	58.3 ± 9.8	< 0.001
FEV_1_, %predicted	87.2 ± 20.2	97.0 ± 14.5	75.1 ± 19.8	< 0.001
MMEF, %predicted	50.4 ± 27.7	68.6 ± 23.1	28.0 ± 11.9	< 0.001
FEF_50_, %predicted	56.4 ± 30.5	76.7 ± 24.2	31.2 ± 14.7	< 0.001
FEF_75_, %predicted	40.7 ± 26.6	55.1 ± 27.1	22.9 ± 9.9	< 0.001
CT parameters				
LAA_-950_, %	2.63 ± 5.36	0.74 ± 1.59	4.96 ± 7.16	< 0.001
LAA_-856_, %	17.0 ± 19.5	7.0 ± 9.9	29.3 ± 21.3	< 0.001
RV, L	2.77 ± 1.03	2.23 ± 0.65	3.43 ± 1.03	< 0.001
TLC, L	5.04 ± 1.17	4.65 ± 1.06	5.52 ± 1.12	< 0.001
IOS parameters				
R5, Ka/L/s	0.35 ± 0.10	0.33 ± 0.09	0.37 ± 0.13	< 0.001
R20, Ka/L/s	0.28 ± 0.07	0.28 ± 0.07	0.27 ± 0.07	0.160
R5–R20, Ka/L/s	0.07 ± 0.07	0.05 ± 0.04	0.09 ± 0.09	< 0.001
X5, Ka/L/s	− 0.12 ± 0.08	− 0.10 ± 0.04	− 0.15 ± 0.10	< 0.001
AX, Ka/L	0.68 ± 0.88	0.39 ± 0.33	1.04 ± 1.17	< 0.001
Fres, Hz	15.1 ± 5.64	13.1 ± 3.7	17.7 ± 6.5	< 0.001

Data are expressed as Mean ± SD or n (%) as appropriate. Differences of characteristics between subjects with and without airflow limitation were compared with the Student’s t test, Mann–Whitney U test, the χ^2^ test, or Fisher exact test as appropriate

*BMI* body mass index, *yrs* years; *mMRC* modified Medical Research Council, *CAT* COPD Assessment Test; *FEV1/FVC* the ratio of forced expiratory volume in the first second to forced vital capacity; *FEV1,%predicted* the ratio of forced expiratory volume in the first second to its predicted value; *MMEF, %predicted* maximal mid-expiratory flow of percent predicted; *FEF*_*50*_*, %predicted and FEF*_*75*_*, %predicted* forced expiratory flow at 50 and 75 of forced vital capacity of percent predicted; *CT* computed tomography; *IOS* impulse oscillometry; *LAA*_*−950*_ low-attenuation area of the lung with attenuation values below -950 Hounsfield units on full-inspiration CT; *LAA-*_*856*_ low-attenuation area of the lung with attenuation values below -856 Hounsfield units on full-expiration CT; *RV* residual volume; *TLC* total lung capacity; *IOS* impulse oscillometry; *R5* resistance at 5 Hz; *R20* resistance at 20 Hz; *R5–R20* the difference from resistance at 5 Hz to resistance at 20 Hz; *X5* reactance at 5 Hz; *AX* reactance area; *Fres* resonant frequency; *%* percent

### Prevalence and risk of SAD increased with aging

The prevalence and the risk of SAD with aging were shown in Tables 2 and 3, respectively. There was a significant linear trend between the prevalence of SAD and advancing ages, irrespective of the methods for the SAD definition (all P for trend < 0.001 for CT-defined SAD, IOS-defined SAD as well as spirometry-defined SAD). But the prevalence varies greatly across different measure methods (ranging from 2.5 on CT to 43.3 on spirometry for subjects at age 49 or younger; ranging from 14.0 on CT to 61.1 on spirometry for subjects at age 50–59; ranging from 36.3 on CT to 82.1 on spirometry for subjects at age 60–69; ranging from 57.3 on CT to 93.2 on spirometry for subjects at age 70 or older). Besides, per decade increase in age was significantly associated with SAD risk after adjusting for possible confounding factors (OR 2.57, 95% CI 2.13 to 3.10, P < 0.001 for CT-defined SAD; OR 1.79, 95% CI 1.54 to 2.08, P < 0.001 for IOS-defined SAD; OR 2.38, 95% CI 2.01 to 2.80, P < 0.001 for spirometry-defined SAD, respectively). When stratified by airflow limitation, all these similar associations (Additional file 1: Tables S1 and S2) were also maintained except for the spirometry-defined SAD in the obstructive group for its extremely high prevalence (830/832), which was possibly owing to the close relationship of value extractions of MMEF%predicted, FEF_50_%predicted or FEF_75_%predicted, and FEV_1_/FVC.

**Table 2:** Prevalence of SAD defined by CT, IOS, and spirometry in total subjects with age stratification

Prevalence (%) of SAD in subgroups stratified by definitional methods	Age (yrs)				P for trend
	≤ 49	50–59	60–69	≥ 70	
Prevalence of SAD defined by CT	2.5 (4/157)	14.0 (83/592)	36.3 (302/831)	57.3 (160/279)	< 0.001
Prevalence of SAD defined by IOS	24.2 (38/157)	21.3 (126/592)	37.4 (311/831)	45.9 (128/279)	< 0.001
Prevalence of SAD defined by spirometry	43.3 (68/157)	61.1 (362/592)	82.1 (682/831)	93.2 (260/279)	< 0.001

P for trend was calculated by linear-by-linear association test. SAD defined by CT was LAA_-856_ > 20%. SAD defined by IOS was R5–R20 > 0.07 Ka/L/s. SAD defined by spirometry was post bronchodilator MMEF %predicted, FEF_50_%predicted or FEF_75_%predicted (any two of the three) < 65%

*SAD* small airway dysfunction, *CT* computed tomography, *IOS* impulse oscillometry, *yrs* years

**Table 3:** Multivariate binary logistic regression analysis of age and SAD risk in total subjects

Variables	Definitional methods for SAD	OR (95% CI)	P value
Age (per 10 yrs increase)	SAD defined by CT	2.57 (2.13–3.10)	< 0.001
	SAD defined by IOS	1.79 (1.54–2.08)	< 0.001
	SAD defined by spirometry	2.38 (2.01–2.80)	< 0.001

CT-defined SAD was defined as LAA_-856_ > 20%. IOS-defined SAD was defined as R5–R20 > 0.07 Ka/L/s. Spirometry-defined SAD was defined as post-bronchodilator MMEF %predicted, FEF_50_%predicted or FEF_75_%predicted (any two of the three) < 65%. All these models were adjusted for sex, BMI, smoking status, smoking index, educational level, asthma, tuberculosis, chronic bronchitis, annual household income, smokers living at home, parental history of respiratory disease, occupation exposure > 6 months, indoor exposure to biomass for cooking or heating. All the variables of age in these models indicate per 10 years increase

*SAD* small airway dysfunction, *CT* computed tomography, *IOS* impulse oscillometry, *%* percent, *OR* odds ratio, *CI* confidence interval, *yrs* years

### Associations of SAD markers and age

**Fig. 2:**
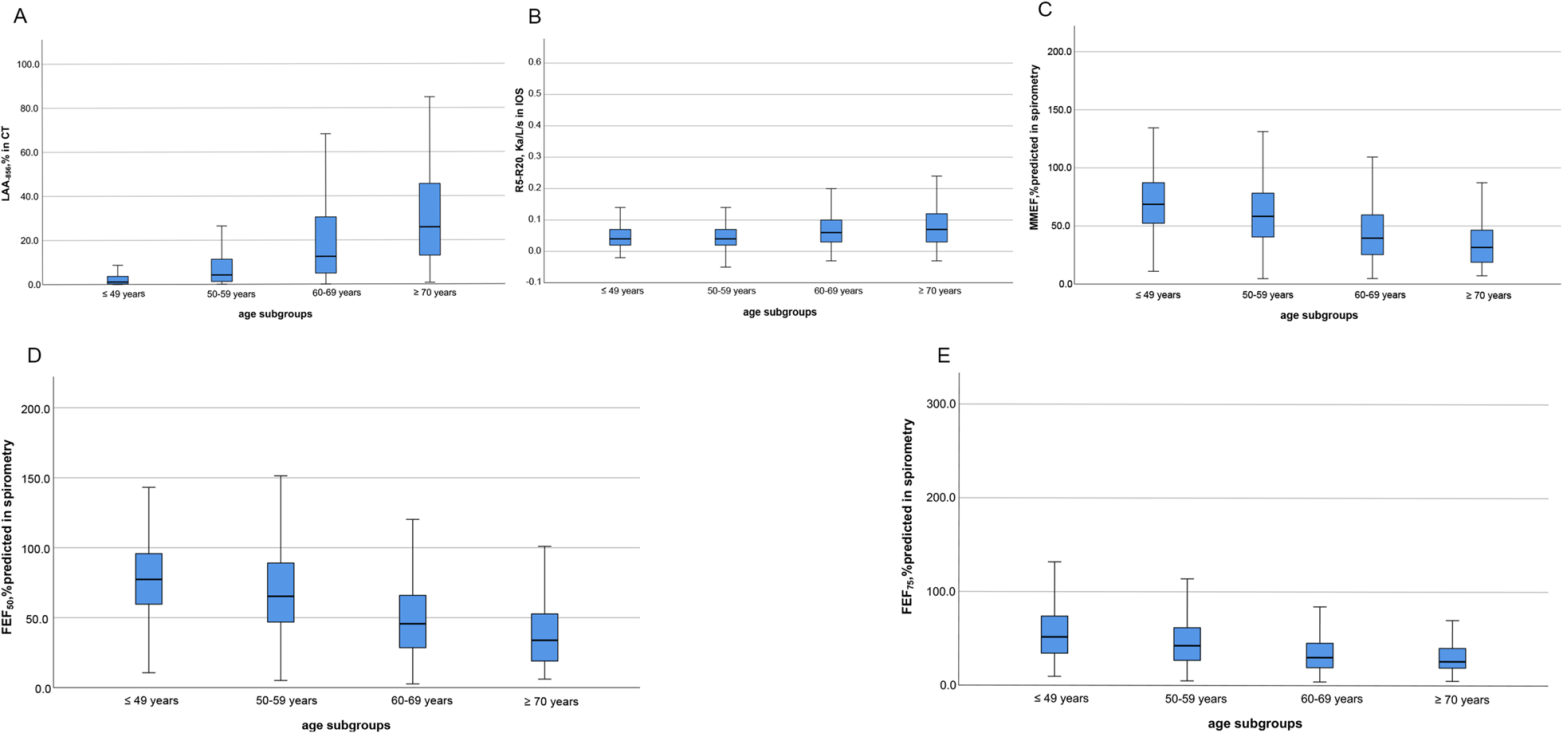
Distribution of small airway abnormality indicated by markers from CT, IOS and spirometry with age stratification in total subjects. Panel **A** was for LAA_·856_ from CT. Panel **B** was for R5–R20 from IOS. Panel **C** was for MMEF, %predicted from spirometry. Panel **D** was for FEF_·50_, %predicted from spirometry. Panel **E** was for FEF_·75_, %predicted from spirometry. Note: Abbreviations: LAA-_856_ = low-attenuation area of the lung with attenuation values below -856 Hounsfield units on full-expiration CT; R5–R20 = the difference from resistance at 5 Hz to resistance at 20 Hz; MMEF, %predicted = maximal mid-expiratory flow of percent predicted; FEF_50_, %predicted and FEF_75_, %predicted = forced expiratory flow at 50 and 75 of forced vital capacity of percent predicted

Overall, subjects in advancing age subgroups tended to have more air trapping (higher LAA_-856_), airway resistance (higher R5–R20), and lower mid-to-end expiratory flow rate (fewer MMEF%predicted, FEF_50_%predicted and FEF_75_%predicted) (Fig. 2). Additionally, the severity of SAD indicated by SAD markers in obstructive subjects is worse than in nonobstructive subjects after stratification. Tentatively, it can be visually seen that the linear trend for LAA_-856_ in CT was more pronounced than other markers (Additional file 1: Fig. S2).

### Contributions of aging to the severity of SAD

**Fig. 3:**
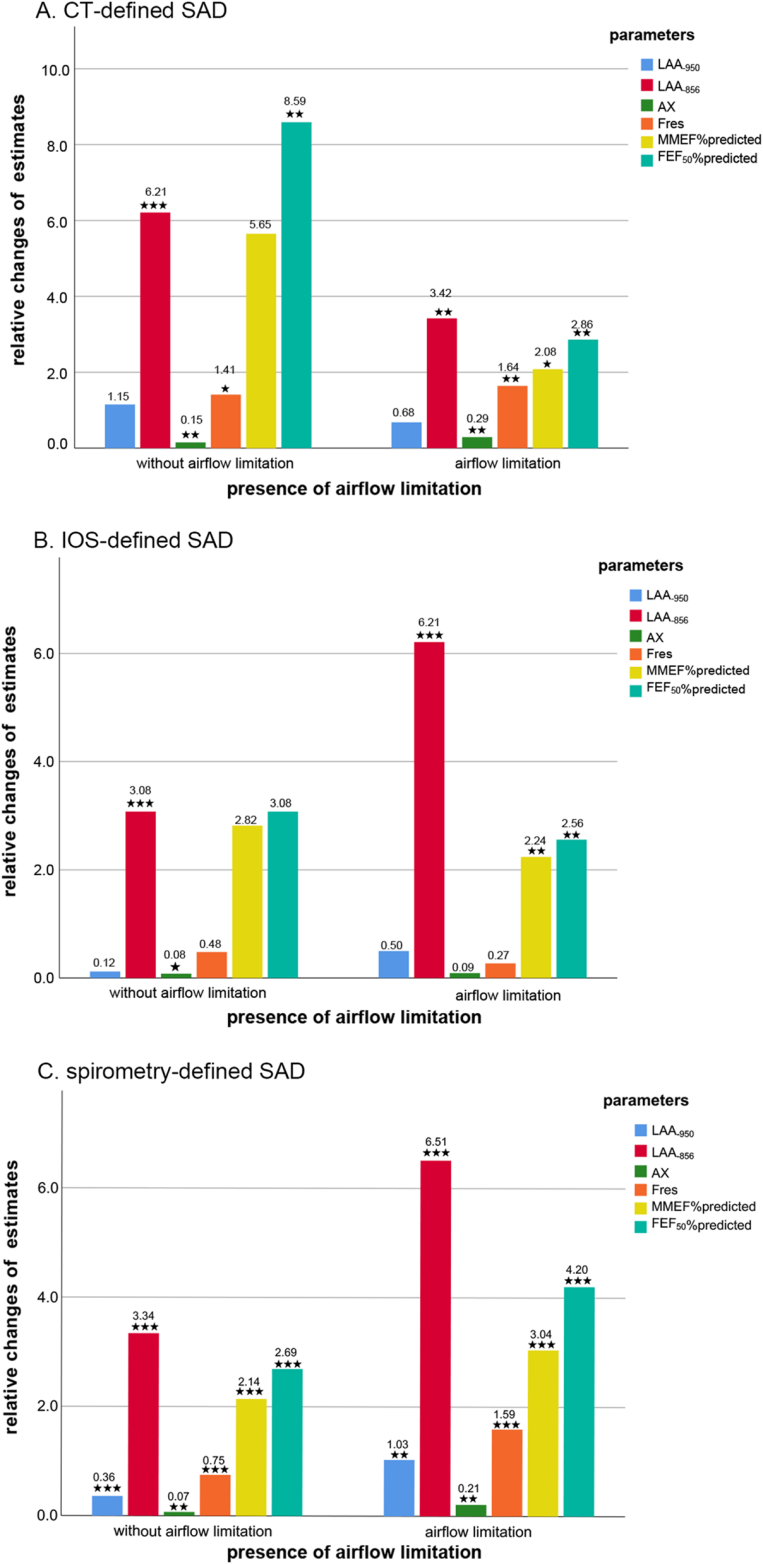
The relative effects of age on the severity of SAD (indicated by markers from CT, IOS and spirometry parameters) in SAD subjects with and without airflow limitation. Note: Panel **A** was for SAD defined by CT. Panel **B** was for SAD defined by IOS. Panel **C** was for SAD defined by spirometry. The relative changes of estimates were abstracted from the Unstandardized β estimates of the multivariate regression models and transformed to their absolute values. These changes are caused by the age increasing per 10 years. *P value < 0.05, **P value < 0.01, ***P value < 0.001. Definitions of abbreviations see Table 1

We analyzed the impact of age on the severity of SAD in the multivariate models adjusted for the same confounders mentioned above. We found that advancing age (per 10 years increase) was significantly associated with 2.09% increase in LAA_-856_ (95% CI − 0.06 to 4.29, P = 0.057 with borderline significance), 0.02 Ka/L/s decrease in X5 (95% CI − 0.04 to − 0.01, P = 0.002), 0.30 Ka/L decrease in AX (95% CI 0.13 to 0.47, P < 0.001), 1.75 Hz increase in Fres (95% CI 0.85 to 2.66, P < 0.001), 3.95% decrease in MMEF %predicted (95% CI − 6.19 to − 1.71, P = 0.001), and 5.42% decrease in FEF50%predicted (95% CI − 7.88 to − 2.95, P < 0.001) in SAD subjects assessed by CT (Table 4). The similar associations mentioned above still persisted in SAD defined by IOS or spirometry (Tables 5 and 6, respectively). Even stratified by airflow limitation, the repeated analyses did not change our results among all the subgroups (Additional file 1: Tables S3, S4, S5, S6, S7, and S8). To our surprise, of all metrics, the parameter with the largest contribution to the severity of SAD almost pointed towards LAA_-856_, an indicator for measuring the degree of air trapping, in all the subgroups for stratification of airflow limitation and definitional methods (Fig. 3). Meanwhile, aging did not have a significant effect on LAA_-950_, which is often used to assess emphysema. However, this was not always the case. The relationship between LAA_-950_ and aging became significant in spirometry-defined SAD subjects regardless of airflow limitation (β 0.36, 95% CI 0.21 to 0.52, P < 0.001 for SAD subjects without airflow limitation; β 1.03, 95% CI 0.36 to 1.70, P = 0.003 for SAD subjects with airflow limitation) (Additional file 1: Table S7, S8 and Fig. S3). Besides, it was also found that the influence of age on AX or Fres always significantly far exceeded that on R5–R20 among all the subgroups. Though R5–R20 is tended to represent peripheral small resistance and is more often to be used in defining SAD.

**Table 4:** Multi-adjusted contributions of age to the severity of SAD among CT-defined SAD subjects (n = 549) from total subjects

Outcomes for SAD markers	Unstandardized β	Standardized β	95% CI	P value
CT				
LAA_−950_, %	0.95	0.08	− 0.06,1.95	0.064
LAA_−856_, %	2.09	0.08	− 0.06, 4.25	0.057
RV, L	− 0.11	− 0.07	− 0.23, 0.02	0.106
TLC, L	− 0.26	− 0.16	− 0.39, − 0.14	< 0.001
IOS				
R5, Ka/L/s	0.02	0.10	0.00, 0.04	0.021
R20, Ka/L/s	0.00	0.004	− 0.00, 0.01	0.918
R5–R20, Ka/L/s	0.02	0.13	0.01, 0.03	0.003
X5, Ka/L/s	− 0.02	− 0.14	− 0.04, − 0.01	0.002
AX, Ka/L	0.30	0.16	0.13, 0.47	< 0.001
Fres, Hz	1.75	0.17	0.85, 2.66	< 0.001
Postbronchodilator				
MMEF, %predicted	− 3.95	− 0.15	− 6.19, − 1.71	0.001
FEF_50_, %predicted	− 5.42	− 0.19	− 7.88, − 2.95	< 0.001
FEF_75_, %predicted	− 0.75	− 0.03	− 2.99, 1.49	0.512

All the models were adjusted for sex, BMI, smoking status, smoking index, educational level, asthma, tuberculosis, chronic bronchitis, annual household income, smokers living at home, parental history of respiratory disease, occupation exposure > 6 months, indoor exposure to biomass for cooking or heating. All the variables of age in these models indicate per 10 years increase

*CI* confidence interval; *β* estimate; % percent; definitions of other abbreviations see Table 1

**Table 5:** Multi-adjusted contributions of age to the severity of SAD among IOS-defined SAD subjects (n = 603) from total subjects

Outcomes for SAD markers	Unstandardized β	Standardized β	95% CI	P value
CT				
LAA_−950_, %	0.64	0.07	− 0.07, 1.34	0.078
LAA_−856_, %	6.12	0.21	4.13, 8.14	< 0.001
RV, L	0.16	0.11	0.05, 0.26	0.003
TLC, L	− 0.17	− 0.11	− 0.28, − 0.07	0.001
IOS				
R5, Ka/L/s	0.01	0.04	− 0.01, 0.02	0.382
R20, Ka/L/s	− 0.00	− 0.01	− 0.01, 0.01	0.755
R5–R20, Ka/L/s	0.01	0.07	− 0.00, 0.01	0.116
X5, Ka/L/s	− 0.02	− 0.16	− 0.03, − 0.01	< 0.001
AX, Ka/L	0.18	0.12	0.06,0.30	0.004
Fres, Hz	0.80	0.14	0.32,1.29	0.001
Postbronchodilator				
MMEF, %predicted	− 6.17	− 0.22	− 8.20, − 4.13	< 0.001
FEF_50_, %predicted	− 7.01	− 0.22	− 9.31, − 4.72	< 0.001
FEF_75_, %predicted	− 4.02	− 0.15	− 6.20, − 1.84	< 0.001

All the models were adjusted for sex, BMI, smoking status, smoking index, educational level, asthma, tuberculosis, chronic bronchitis, annual household income, smokers living at home, parental history of respiratory disease, occupation exposure > 6 months, indoor exposure to biomass for cooking or heating. All the variables of age in these models indicate per 10 years increase. Definitions of abbreviations see Table 1

**Table 6:** Multi-adjusted contributions of age to the severity of SAD among spirometry-defined SAD subjects (n = 1372) from total subjects

Outcomes for SAD markers	Unstandardized β	Standardized β	95% CI	P value
CT				
LAA_−950_, %	1.03	0.13	0.63, 1.43	< 0.001
LAA_−856_, %	6.86	0.26	5.56, 8.16	< 0.001
RV, L	0.17	0.12	0.10, 0.23	< 0.001
TLC, L	− 0.17	− 0.12	− 0.24, − 0.10	< 0.001
IOS				
R5, Ka/L/s	0.02	0.10	0.01, 0.02	< 0.001
R20, Ka/L/s	0.00	− 0.001	− 0.01, 0.004	0.964
R5–R20, Ka/L/s	0.02	0.15	0.01, 0.02	< 0.001
X5, Ka/L/s	− 0.02	− 0.16	− 0.02, − 0.01	< 0.001
AX, Ka/L	0.21	0.16	0.14, 0.28	< 0.001
Fres, Hz	1.60	0.21	1.18, 2.03	< 0.001
Postbronchodilator				
MMEF, %predicted	− 5.32	− 0.26	− 6.40, − 4.24	< 0.001
FEF_50_, %predicted	− 6.77	− 0.27	− 8.08, − 5.46	< 0.001
FEF_75_, %predicted	− 2.40	− 0.14	− 3.33, − 1.46	< 0.001

All the models were adjusted for sex, BMI, smoking status, smoking index, educational level, asthma, tuberculosis, chronic bronchitis, annual household income, smokers living at home, parental history of respiratory disease, occupation exposure > 6 months, indoor exposure to biomass for cooking or heating. All the variables of age in these models indicate per 10 years increase. Definitions of abbreviations see Table 1

## Discussion

In our study, we identified several novel findings. First, aging is significantly associated with the prevalence, the increased risk, and the worse severity of SAD. Second, the greatest effect of aging on the severity of SAD is indicated by air trapping of LAA_-856_, which suggests that CT may be the optimum diagnostic method for detecting SAD accurately. Third, aging has a more pronounced effect on AX and Fres rather than R5–R20.

Several previous studies have reported on the prevalence of SAD. However, the prevalence varied greatly, ranging from 6.7% in veterans during the 1990–1991 Gulf War [27] to 53.8% in physician-diagnosed asthmatic patients [28]. The difference may result from various spirometry diagnostic criteria used and the various targeted population. Of these, the most well-represented prevalence of SAD for Chinese was reported by Xiao et al. [26] They demonstrated that the overall prevalence was 46.9%, which increased from 21.5% at age 20–29 to 74.7% at age 70 or older. And the prevalence of SAD in our study was similar, although the diagnostic criteria they used was the pre-bronchodilator lung function testing. The difference may result from a targeted number of 1000 COPD patients and 1000 non-COPD participants in the recruitment (COPD subjects comprise roughly one-half of the total subjects), which caused a higher prevalence of SAD than that in the general population reported by Xiao et al. [26].

We further explored the prevalence of SAD diagnosed by CT and IOS. Surprisingly, the prevalence diagnosed by CT or IOS remains increasing with aging and is much lower than that diagnosed by spirometry. It may be due to the characteristics of the various measurement methods which detected different subtypes of SAD. After adjusting for possible risk factors for SAD, aging is still significantly associated with the risk of SAD. It may be suggested that we couldn’t simply view age as a comprehensive word. For example, environmental toxic factors, and insults exposure (such as cigarette, biomass and pollution exposure) increase with aging [28]. We couldn’t simply argue that the aging lung changes in SAD may be directly resulted from an accumulation of environmental factors and indirectly mediated by age, and completely neglected the effects of age itself. In contrast, what we found in our study reminds us also to pay more attention to the role of age itself on lung structural and functional changes. As for the underlying mechanisms of aging on SAD, the exact truth remains unknown. And Brandenberger et al. found the possible answers may come from various types of pulmonary resident cells and the immune system associated with aging [29].

One of the most important findings in our study is that advancing age was associated with the increase in the severity of air trapping (LAA_-856_) but not emphysema (LAA_-950_) in SAD defined by CT or IOS. This is similar to what was found by Martinez et al. in subjects without obstruction or respiratory impairment. He described that PRM^FSA^ increased significantly by 2.7% per decade, but PRM^EMPH^ increased non-significantly (P = 0.34) [10]. We reasonably speculated a possible explanation for this phenomenon. It has been demonstrated that airspace enlargement precedes emphysema in senile lungs [30]. Besides, a review in *Chest* journal also concluded that morphologic changes in the normal aging lung consisted of alveolar enlargement but without wall destruction and distal duct ectasia [31]. More convincingly, McDonough et al. did identify small conducting airway abnormalities before the onset of emphysematous destruction using micro-CT [7]. This phenomenon can also be distinguished using PRM^fSAD^ even on high-resolution CT [32]. Although there is a little difference between the concept of PRM^fSAD^ and LAA_-856_, they all represent small airway dysfunction to some extent.

Besides, different from previous studies, we additionally evaluated the effects of aging on the severity of SAD by IOS and spirometry. After comparisons across various SAD markers of the 3 methods, LAA_-856_ on CT is the most sensitive indicator to identify the age-related alterations in SAD. Previously, there has been a long-standing debate that which method (CT, IOS, and spirometry) provides the most reliable and available measure for SAD [11, 33]. Our research provides some evidence for the preferred choice of CT methods when assessing SAD. Different from SAD defined by CT and IOS, aging contributes significantly to the emphysema index (LAA_-950_) in the severity of SAD if SAD was defined by spirometry. It may also prove reversely that CT could identify SAD in an earlier phrase (before emphysema construction appears) than spirometry.

To the best of our knowledge, the R5–R20 criterion in IOS was common to define SAD in asthma patients [34]. And now some studies directly copied this criterion to assess SAD in COPD without evidence and ignoring the values of other parameters on IOS. We found that AX or Fres contributed greater to the severity of SAD with age per 10 years increase than R5–R20 in a community-based population. This is consistent with some previous studies. For example, when detecting SAD in symptomatic patients with preserved pulmonary function, AX and Fres have greater diagnostic power (greater AUC value) than R5–R20 [35, 36]. Hence, it may be not reasonable to directly use R5–R20 to diagnose SAD in a general or COPD population. And we should attach more importance to AX or Fres with possible greater potential value in assessing SAD in non-asthma. But the cutoff values, mechanisms, and practical applications behind them need to be validated further in the future.

Our study was the first to comprehensively evaluate the relationship between aging and SAD across CT, IOS, and spirometry measures in Chinese with the relative large sample size. What we found not only confirmed that SAD is really an abnormality associated with aging, but also indicated that CT may be the first preferred measure to assess SAD in future studies. Moreover, it is also suggested that we should attach more importance to AX or Fres markers than R5–R20 when researching values of the IOS test in detecting SAD in COPD.

Our study is also subject to several limitations. First, SAD was defined by LAA_-856_ instead of PRM^fSAD^, which may be more powerful in diagnosing functional SAD. But the slight difference did not affect our conclusions consistent with the previous findings. Second, our study was only a cross-sectional study. Lacking longitudinal data hinders us from longitudinally extending the effects of age progression on SAD and validating whether LAA_-856_ remains most sensitive in a long-time and intra-individual assessment. But we have tried our best to adjust all the possible risk factors for SAD to minimize inter-individual variance. Third, the prevalence of SAD detected by CT in subjects below 50 may be interpreted with caution not only because of the relatively small numbers but also for the age of these subjects in this group mainly focusing on 40–50 years old (Subjects among 40–50 years old were composed of 93% in these 157 subjects approximately). Hence, these results might not be generalized to a younger population below 40. It may result from the fact that our cohort mainly recruited subjects with 40–80 years old at the enrollment, which are also the currently recommended screening ages for lung health checkups. Moreover, most subjects below 50 are probably healthier than a random sample, as well as less willing to participate in such studies. Despite this shortcoming, it indeed reflects the true characteristics of this population needing health checkups. Finally, participants in ECOPD were volunteers and were recruited with a targeted number of about 1000 participants with COPD and about 1000 without COPD, not fully representative of a general population, causing a little higher prevalence of SAD. However, to make our results more powerful, we repeated all the analyses in these subjects stratified by airflow limitation and elaborated the relationship further between aging and SAD outside the influence of airflow limitation.

## Conclusions

Aging was significantly associated with the increase in the prevalence, risk, and severity of SAD, of which the greatest effect was indicated by LAA_-856_ on CT. These findings not only suggest that it’s essential to emphasize the effects of age on early lung disease in future research, but also indicate that CT may be the most optimal measure to detect the age-related changes for SAD. Besides, the traits of AX and Fres in SAD population of various age shed new light on considering values of AX or Fres parameters when assessing SAD with IOS in the future. Future studies are required to determine the molecular mechanisms of aging in early lung diseases such as SAD as well as the practical applications of CT or AX and Fres in IOS in detecting SAD.

## Supplementary Information

**Additional file 1: Table S1.** Prevalence of SAD defined by CT, IOS, and spirometry over age in subjects with and without airflow limitation. **Table S2.** Multivariate binary logistic regression analysis of age and SAD risk in subjects with and without airflow limitation. **Table S3.** Multi-adjusted contributions of age to the severity of SAD among CT-defined SAD subjects from subjects without airflow limitation (n = 81). **Table S4.** Multi-adjusted contributions of age to the severity of SAD among CT-defined SAD subjects from subjects with airflow limitation (n = 468). **Table S5.** Multi-adjusted contributions of age to the severity of SAD among IOS-defined SAD subjects from subjects without airflow limitation (n = 207). **Table S6.** Multi-adjusted contributions of age to the severity of SAD among IOS-defined SAD subjects from subjects with airflow limitation (n = 396). **Table S7.** Multi-adjusted contributions of age to the severity of SAD among spirometry-defined SAD subjects from subjects without airflow limitation (n = 542). **Table S8.** Multi-adjusted contributions of age to the severity of SAD among spirometry-defined SAD subjects from subjects with airflow limitation (n = 830). **Fig. S1.** Flow diagram for stratification of 1859 subjects by airflow limitation and definitional methods. **Fig. S2.** Distribution of small airway abnormality indicated by markers from CT, IOS and spirometry over age stratification in subjects with and without airflow limitation. 

## Data Availability

The datasets used and analyzed in this study are available from the corresponding author on reasonable requests.

## Abbreviations

SAD: Small airway dysfunction
CT: Computed tomography
IOS: Impulse oscillometry
COPD: Chronic obstructive pulmonary disease
ECOPD: The early COPD
FEV_1_: Forced expiratory volume in the first second
FEV_1_, %Predicted: The ratio of FEV_1_ to its predicted value
FEV_1_/FVC: The ratio of FEV_1_ to forced expiratory volume
MMEF, %Predicted: Maximal mid-expiratory flow of percent predicted
FEF_50_, %predicted and FEF_75_, %predicted: Forced expiratory flow at 50 and 75 of forced vital capacity of percent predicted
LAA-_950_: Low-attenuation area of the lung with attenuation values below -950 Hounsfield units on full inspiration CT
LAA_-856_: Low-attenuation area of the lung with attenuation values below -856 Hounsfield units on full-expiration CT
RV: Residual volume
TLC: Total lung capacity
R5: Resistance at 5 Hz
R20: Resistance at 20 Hz
R5–R20: The difference from R5 to R20
X5: Reactance at 5 Hz
AX: Reactance area
Fres: Resonant frequency
CI: Confidence interval
PRM: Parametric response map
