# Supplementary material for: The association between small airway dysfunction and aging: a cross-sectional analysis from the ECOPD cohort
Source: Respir Res. 2022 Sep 4;23:229. doi: 10.1186/s12931-022-02148-w (PMC9441095; doi:10.1186/s12931-022-02148-w)
Supplement: Supplementary file 1 — Additional file 1: Table S1. Prevalence of SAD defined by CT, IOS, and spirometry over age in subjects with and without airflow limitation. Table S2. Multivariate binary logistic regression analysis of age and SAD risk in subjects with and without airflow limitation. Table S3. Multi-adjusted contributions of age to the severity of SAD among CT-defined SAD subjects from subjects without airflow limitation (n = 81). Table S4. Multi-adjusted contributions of age to the severity of SAD among CT-defined SAD subjects from subjects with airflow limitation (n = 468). Table S5. Multi-adjusted contributions of age to the severity of SAD among IOS-defined SAD subjects from subjects without airflow limitation (n = 207). Table S6. Multi-adjusted contributions of age to the severity of SAD among IOS-defined SAD subjects from subjects with airflow limitation (n = 396). Table S7. Multi-adjusted contributions of age to the severity of SAD among spirometry-defined SAD subjects from subjects without airflow limitation (n = 542). Table S8. Multi-adjusted contributions of age to the severity of SAD among spirometry-defined SAD subjects from subjects with airflow limitation (n = 830). Fig. S1. Flow diagram for stratification of 1859 subjects by airflow limitation and definitional methods. Fig. S2. Distribution of small airway abnormality indicated by markers from CT, IOS and spirometry over age stratification in subjects with and without airflow limitation. [file 12931_2022_2148_MOESM1_ESM.docx]

**The association between small airway dysfunction and aging: a cross-sectional analysis from the ECOPD cohort.**

Cuiqiong Dai^1^*; Fan Wu^1 2^*; Zihui Wang^1^*; Jieqi Peng^1^*; Huajing Yang^1^; Youlan Zheng^1^; Lifei Lu^1^; Ningning Zhao^1^; Zhishan Deng^1^; Shan Xiao^1^; Xiang Wen^1^; Jianwu Xu^1^; Peiyu Huang^1^; Kunning Zhou^1^; Xiaohui Wu^1^; Yumin Zhou^1 2#^; Pixin Ran^1 2#^

**Supplemental materials**

**Statistical analyses**

Data regarding characteristics are expressed as mean ± standard deviation. We made univariate comparisons between the two groups concerning characteristics by using Student’s t test for continuous variables with normal distribution or Mann-Whitney U test for continuous variables with skewness distribution, and the χ² test or Fisher exact test for categorical variables respectively. The linear-by-linear association test was conducted for the prevalence of SAD and the polynomial linear trend test for SAD markers across various age stratification. We applied multivariate binary logistic regression models to assess the association of age with SAD risk. Multiple linear regression models were carried out to determine the impact of age on the severity of SAD in SAD subjects. We conducted all the above analyses in total subjects and then repeated them in the subjects with the stratification of airflow limitation. Finally, to intuitively compare the contributions of age to a variety of SAD markers, we extracted parameters from CT, IOS, and spirometry according to the largest or the second-largest absolute estimates of their multiple linear regression models among the 6 stratified subgroups, and then visualized these parameters in a vertical bar. Covariates adjusted in all the regression models were sex, BMI, smoking status, smoking index, educational level, asthma, tuberculosis, chronic bronchitis, annual household income, smokers living at home, parental history of respiratory diseases, occupational exposure > 6 months, indoor exposure to biomass for cooking or heating. There was no imputation in our study for no missing data on the main variables of interest. All the data analyses were performed on IBM SPSS Version 25.0. Two-sided p values less than 0.05 were considered statistically significant.

**Supplemental tables**

Table S1. Prevalence of SAD defined by CT, IOS, and spirometry over age in subjects with and without airflow limitation.

| variables | Subgroups stratified by airflow limitation and definitonal methods | | Age stratification(yrs) | | | | P for trend |
| --- | --- | --- | --- | --- | --- | --- | --- |
|  |  |  | <= 49 | 50-59 | 60-69 | >= 70 |  |
| Prevalence (%) of SAD | subjects without airflow limitation | SAD defined by CT | 0.70 (1/139) | 4.9 (21/426) | 9.2 (35/381) | 29.6 (24/81) | <0.001 |
|  |  | SAD defined by IOS | 20.1 (28/139) | 15.7 (67/426) | 23.9 (91/381) | 25.9 (21/81) | 0.025 |
|  |  | SAD defined byspirometry | 36.0 (50/139) | 46.2 (197/426) | 61.2 (233/381) | 76.5 (62/81) | <0.001 |
|  | subjects with airflow limitation | SAD defined by CT | 16.7 (3/18) | 37.3 (62/166) | 59.3 (267/450) | 68.7 (136/198) | <0.001 |
|  |  | SAD defined by IOS | 55.6 (10/18) | 35.5 (59/166) | 48.9 (220/450) | 54.0 (107/198) | 0.004 |
|  |  | SAD defined by spirometry | 100 (18/18) | 99.4(165/166) | 99.8(449/450) | 100(198/198) | 0.333 |

Note: P for trend was calculated by linear-by-linear association test. SAD defined by CT was LAA_-856_ > 20%. SAD defined by IOS was R5-R20 > 0.07 Ka/L/s. SAD defined by spirometry was post bronchodilator MMEF %predicted, FEF_50_ %predicted or FEF_75_ %predicted (any two of the three) < 65%. SAD = small airway dysfunction, CT = computed tomography, IOS = impulse oscillometry, yrs = years, % = percent.

Table S2. Multivariate binary logistic regression analysis of age and SAD risk in subjects with and without airflow limitation.

| variables | Subgroups | definitional methods for SAD | OR (95%CI) | P value |
| --- | --- | --- | --- | --- |
| Age (per 10 yrs increase) | Subjects without airflow limitation | SAD defined by CT | 2.78 (1.91-4.03) | <0.001 |
|  |  | SAD defined by spirometry | 1.47 (1.17-1.85) | <0.001 |
|  |  | SAD defined by IOS | 1.79 (1.49-2.14) | <0.001 |
|  | Subjects with airflow limitation | SAD defined by CT | 1.73 (1.35-2.23) | <0.001 |
|  |  | SAD defined by IOS | 1.53 (1.22-1.93) | <0.001 |
|  |  | SAD defined by spirometry | 37.4(0.02-81696.6) | 0.356 |

Note: CT-defined SAD was defined as LAA_-856_>20%. IOS-defined SAD was defined as R5-R20>0.07 Ka/L/s. Spirometry-defined SAD was defined as post bronchodilator MMEF %predicted, FEF50 %predicted or FEF75 %predicted (any two of the three) < 65%. All these models were all adjusted for sex, BMI, smoking status, smoking index, educational level, asthma, tuberculosis, chronic bronchitis, annual household income, smokers living at home, parental history of respiratory disease, occupation exposure > 6 months, indoor exposure to biomass for cooking or heating. All the variables of age in these models indicate per 10 years increase.

Abbreviations: SAD = small airway dysfunction, CT = computed tomography, IOS = impulse oscillometry, OR = odds ratio, CI = confidence interval

Table S3. Multi-adjusted contributions of age to the severity of SAD among CT-defined SAD subjects from subjects without airflow limitation (n = 81).

| Outcomes for SAD markers | Unstandardized β | Standardized β | 95%CI | P value |
| --- | --- | --- | --- | --- |
| CT |  |  |  |  |
| LAA_−950_, % | 1.15 | 0.21 | -0.25, 2.55 | 0.105 |
| LAA_−856_, % | 6.21 | 0.21 | 4.32, 8.10 | <0.001 |
| RV, L | -0.25 | -0.22 | -0.52, 0.02 | 0.074 |
| TLC, L | -0.15 | -0.12 | -0.43, 0.13 | 0.280 |
| IOS |  |  |  |  |
| R5, Ka/L/s | 0.02 | 0.16 | -0.00, 0.04 | 0.113 |
| R20, Ka/L/s | 0.01 | 0.08 | -0.01, 0.02 | 0.383 |
| R5-R20, Ka/L/s | 0.01 | 0.18 | -0.00, 0.02 | 0.155 |
| X5, Ka/L/s | -0.02 | -0.33 | -0.03, -0.00 | 0.011 |
| AX, Ka/L | 0.15 | 0.35 | 0.05, 0.25 | 0.005 |
| Fres, Hz | 1.41 | 0.32 | 0.29, 2.53 | 0.015 |
| postbronchodilator |  |  |  |  |
| MMEF, %predicted | -5.65 | -0.25 | -11.45, 0.14 | 0.056 |
| FEF_50_, %predicted | -8.59 | -0.37 | -14.47, -2.70 | 0.005 |
| FEF_75_, %predicted | -1.94 | -0.06 | -10.39, 6.51 | 0.648 |

All the models were adjusted for sex, BMI, smoking status, smoking index, educational level, asthma, tuberculosis, chronic bronchitis, annual household income, smokers living at home, parental history of respiratory disease, occupation exposure > 6 months, indoor exposure to biomass for cooking or heating.All the variables of age in these models mean per 10 years unit increase.

Abbreviations: CI = confidence interval; β = estimate; definitions of other abbreviations see Table 1.

Table S4. Multi-adjusted contributions of age to the severity of SAD among CT-defined SAD subjects from subjects with airflow limitation (n = 468).

| Outcomes for SAD markers | Unstandardized β | Standardized β | 95%CI | P value |
| --- | --- | --- | --- | --- |
| CT |  |  |  |  |
| LAA_−950_, % | 0.68 | 0.05 | -0.51,1.88 | 0.263 |
| LAA_−856_, % | 3.42 | 0.13 | 0.96,5.89 | 0.007 |
| RV, L | -0.05 | -0.03 | -0.19,0.10 | 0.519 |
| TLC, L | -0.32 | -0.19 | -0.47,-0.17 | <0.001 |
| IOS |  |  |  |  |
| R5, Ka/L/s | 0.02 | 0.07 | -0.01,0.03 | 0.136 |
| R20, Ka/L/s | -0.00 | -0.04 | -0.01,0.01 | 0.395 |
| R5-R20, Ka/L/s | 0.02 | 0.12 | 0.00,0.03 | 0.015 |
| X5, Ka/L/s | -0.02 | -0.12 | -0.04,-0.00 | 0.016 |
| AX, Ka/L | 0.29 | 0.14 | 0.09,0.49 | 0.004 |
| Fres, Hz | 1.64 | 0.16 | 0.62,2.67 | 0.002 |
| postbronchodilator |  |  |  |  |
| MMEF, %predicted | -2.08 | -0.12 | -3.78,-0.39 | 0.016 |
| FEF_50_, %predicted | -2.86 | -0.15 | -4.76,-0.96 | 0.003 |
| FEF_75_, %predicted | 0.19 | 0.01 | -1.37,1.75 | 0.813 |

Note: All the models were adjusted for sex, BMI, smoking status, smoking index, educational level, asthma, tuberculosis, chronic bronchitis, annual household income, smokers living at home, parental history of respiratory disease, occupation exposure > 6 months, indoor exposure to biomass for cooking or heating.All the variables of age in these models mean per 10 years unit increase.

Abbreviations: CI = confidence interval; β = estimate; definitions of other abbreviations see Table 1.

Table S5. Multi-adjusted contributions of age to the severity of SAD among IOS-defined SAD subjects from subjects without airflow limitation (n = 207).

| Outcomes for SAD markers | Unstandardized β | Standardized β | 95%CI | P value |
| --- | --- | --- | --- | --- |
| CT |  |  |  |  |
| LAA_−950_, % | 0.12 | 0.13 | -0.00,0.25 | 0.056 |
| LAA_−856_, % | 3.08 | 0.38 | 1.97,4.19 | <0.001 |
| RV, L | 0.09 | 0.15 | 0.01,0.18 | 0.022 |
| TLC, L | -0.12 | -0.10 | -0.25,0.01 | 0.074 |
| IOS |  |  |  |  |
| R5, Ka/L/s | 0.00 | -0.00 | -0.01,0.01 | 0.982 |
| R20, Ka/L/s | -0.00 | -0.04 | -0.01,0.01 | 0.605 |
| R5-R20, Ka/L/s | 0.00 | 0.06 | -0.01,0.01 | 0.406 |
| X5, Ka/L/s | -0.01 | -0.20 | -0.02,-0.00 | 0.006 |
| AX, Ka/L | 0.08 | 0.17 | 0.01,0.15 | 0.022 |
| Fres, Hz | 0.48 | 0.15 | 0.00,0.97 | 0.050 |
| postbronchodilator |  |  |  |  |
| MMEF, %predicted | -2.82 | -0.12 | -6.13,0.50 | 0.095 |
| FEF_50_, %predicted | -3.08 | -0.12 | -6.65,0.49 | 0.090 |
| FEF_75_, %predicted | -1.39 | -0.05 | -5.88,3.11 | 0.543 |

Note: All these models were all adjusted for sex, BMI, smoking status, smoking index, educational level, asthma, tuberculosis, chronic bronchitis, annual household income, smokers living at home, parental history of respiratory disease, occupation exposure > 6 months, indoor exposure to biomass for cooking or heating. All the variables of age in these models indicate per 10 years increase. Abbreviations: CI = confidence interval; β = estimate; definitions of other abbreviations see Table 1.

Table S6. Multi-adjusted contributions of age to the severity of SAD among IOS-defined SAD subjects from subjects with airflow limitation (n = 396).

| Outcomes for SAD markers | Unstandardized β | Standardized β | 95%CI | P value |
| --- | --- | --- | --- | --- |
| CT |  |  |  |  |
| LAA_−950_, % | 0.50 | 0.04 | -0.63,1.63 | 0.385 |
| LAA_−856_, % | 6.21 | 0.21 | 4.32,8.10 | <0.001 |
| RV, L | 0.02 | 0.02 | -0.12,0.17 | 0.758 |
| TLC, L | -0.32 | -0.20 | -0.47,-0.16 | <0.001 |
| IOS |  |  |  |  |
| R5, Ka/L/s | -0.00 | -0.02 | -0.02,0.01 | 0.668 |
| R20, Ka/L/s | -0.01 | -0.05 | -0.01,0.00 | 0.287 |
| R5-R20, Ka/L/s | 0.00 | 0.01 | -0.01,0.01 | 0.843 |
| X5, Ka/L/s | -0.01 | -0.09 | 0.12,0.00 | 0.117 |
| AX, Ka/L | 0.09 | 0.05 | -0.10,0.28 | 0.334 |
| Fres, Hz | 0.27 | 0.04 | -0.43,0.98 | 0.442 |
| postbronchodilator |  |  |  |  |
| MMEF, %predicted | -2.24 | -0.08 | -3.59,-0.89 | 0.001 |
| FEF_50_, %predicted | -2.56 | -0.15 | -4.18,-0.95 | 0.002 |
| FEF_75_, %predicted | -0.57 | -0.05 | -1.79,0.64 | 0.354 |

Note: All these models were all adjusted for sex, BMI, smoking status, smoking index, educational level, asthma, tuberculosis, chronic bronchitis, annual household income, smokers living at home, parental history of respiratory disease, occupation exposure > 6 months, indoor exposure to biomass for cooking or heating. All the variables of age in these models indicate per 10 years increase. Abbreviations: CI = confidence interval; β = estimate; definitions of other abbreviations see Table 1.

Table S7. Multi-adjusted contributions of age to the severity of SAD among spirometry-defined SAD subjects from subjects without airflow limitation (n = 542).

| Outcomes for SAD markers | Unstandardized β | Standardized β | 95%CI | P value |
| --- | --- | --- | --- | --- |
| CT |  |  |  |  |
| LAA_−950_, % | 0.36 | 0.19 | 0.21,0.52 | <0.001 |
| LAA_−856_, % | 3.34 | 0.24 | 2.16,4.53 | <0.001 |
| RV, L | 0.08 | 0.09 | 0.01,0.14 | 0.021 |
| TLC, L | -0.13 | -0.10 | -0.21,-0.05 | 0.003 |
| IOS |  |  |  |  |
| R5, Ka/L/s | 0.01 | 0.04 | -0.00,0.01 | 0.263 |
| R20, Ka/L/s | 0.00 | 0.00 | -0.01,0.01 | 0.962 |
| R5-R20, Ka/L/s | 0.01 | 0.09 | 0.00,0.01 | 0.043 |
| X5, Ka/L/s | -0.01 | -0.14 | -0.01,-0.00 | 0.002 |
| AX, Ka/L | 0.07 | 0.14 | 0.03,0.11 | 0.001 |
| Fres, Hz | 0.75 | 0.15 | 0.34,1.17 | <0.001 |
| postbronchodilator |  |  |  |  |
| MMEF, %predicted | -2.14 | -0.17 | -3.25,-1.03 | <0.001 |
| FEF_50_, %predicted | -2.69 | -0.17 | -4.08,-1.29 | <0.001 |
| FEF_75_, %predicted | -0.35 | -0.02 | -1.64,0.94 | 0.592 |

Note: All these models were all adjusted for sex, BMI, smoking status, smoking index, educational level, asthma, tuberculosis, chronic bronchitis, annual household income, smokers living at home, parental history of respiratory disease, occupation exposure > 6 months, indoor exposure to biomass for cooking or heating. All the variables of age in these models indicate per 10 years increase. Abbreviations: CI = confidence interval; β = estimate; definitions of other abbreviations see Table 1.

Table S8. Multi-adjusted contributions of age to the severity of SAD among spirometry-defined SAD subjects from subjects with airflow limitation (n = 830).

| Outcomes for SAD markers | Unstandardized β | Standardized β | 95%CI | P value |
| --- | --- | --- | --- | --- |
| CT |  |  |  |  |
| LAA_−950_, % | 1.03 | 0.10 | 0.36,1.70 | 0.003 |
| LAA_−856_, % | 6.51 | 0.22 | 4.57,8.45 | <0.001 |
| RV, L | 0.09 | 0.06 | -0.01,0.18 | 0.088 |
| TLC, L | -0.31 | -0.20 | -0.41,-0.20 | <0.001 |
| IOS |  |  |  |  |
| R5, Ka/L/s | 0.01 | 0.06 | -0.00,0.02 | 0.112 |
| R20, Ka/L/s | -0.01 | -0.05 | -0.01,0.00 | 0.120 |
| R5-R20, Ka/L/s | 0.02 | 0.12 | 0.01,0.02 | 0.001 |
| X5, Ka/L/s | -0.02 | -0.14 | -0.03,-0.01 | <0.001 |
| AX, Ka/L | 0.21 | 0.13 | 0.09,0.33 | 0.001 |
| Fres, Hz | 1.59 | 0.17 | 0.92,2.25 | <0.001 |
| postbronchodilator |  |  |  |  |
| MMEF, %predicted | -3.04 | -0.18 | -4.23,-1.84 | <0.001 |
| FEF_50_, %predicted | -4.20 | -0.20 | -5.62,-2.78 | <0.001 |
| FEF_75_, %predicted | -0.65 | -0.05 | -1.68,0.39 | 0.218 |

Note: All these models were all adjusted for sex, BMI, smoking status, smoking index, educational level, asthma, tuberculosis, chronic bronchitis, annual household income, smokers living at home, parental history of respiratory disease, occupation exposure > 6 months, indoor exposure to biomass for cooking or heating. All the variables of age in these models indicate per 10 years increase. Abbreviations: CI =confidence interval; β = estimate; definitions of other abbreviations see Table 1.

**Supplemental figures**

Figure S1. Flow diagram for stratification of 1859 subjects by airflow limitation and definitional methods.


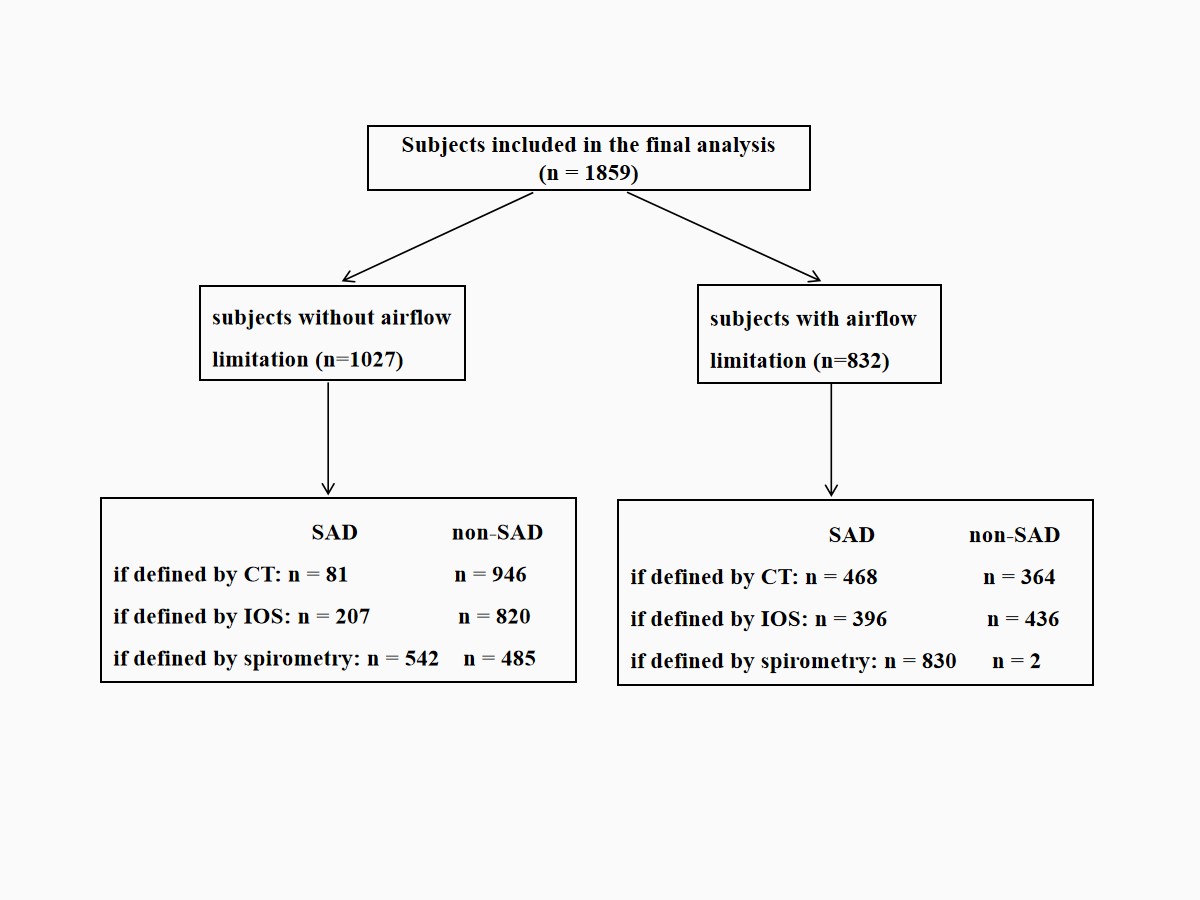


Note: SAD = small airway dysfunction, CT = computed tomography, IOS = impulse oscillometry

Figure S2. Distribution of small airway abnormality indicated by markers from CT, IOS and spirometry over age stratification in subjects with and without airflow limitation.


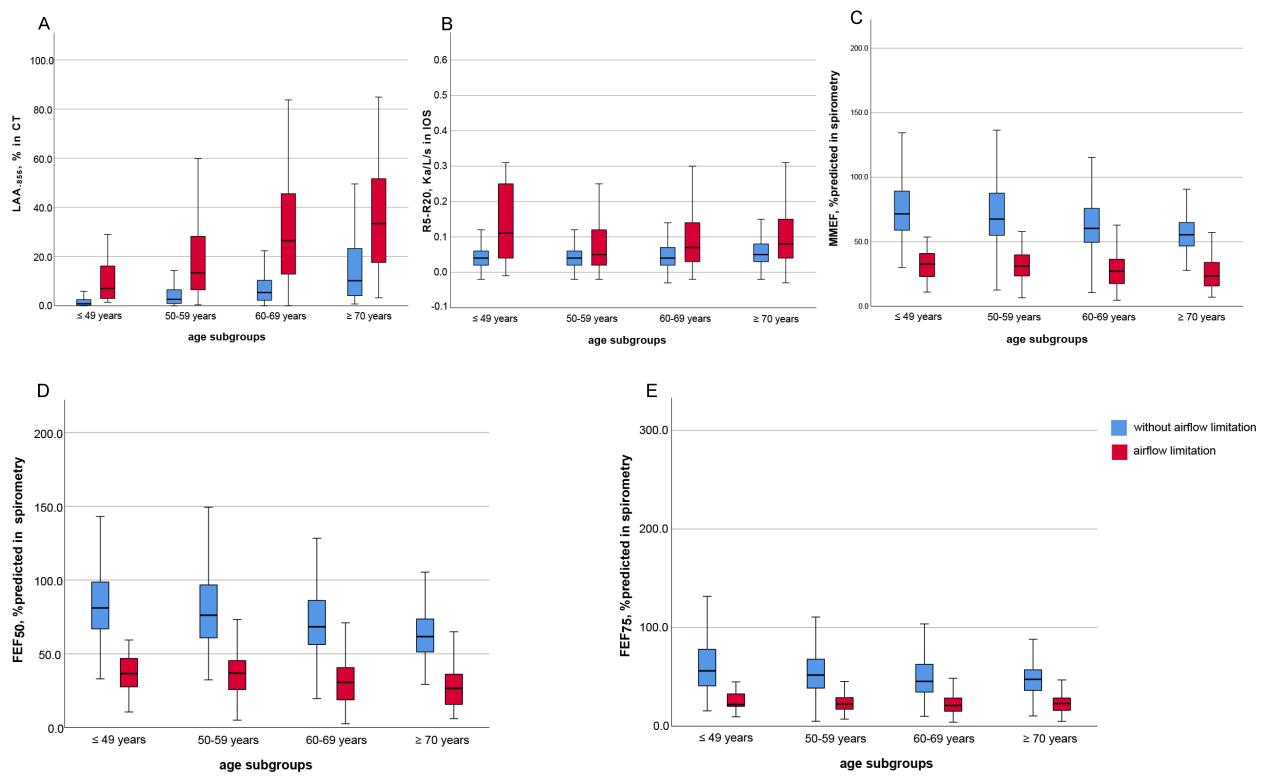


Note: Panel A was for LAA_-856_ from CT. Panel B was for R5–R20 from IOS. Panel C was for MMEF, %predicted from spirometry. Panel D was for FEF_50_, %predicted from spirometry. Panel E was for FEF_75_, %predicted from spirometry. Abbreviations: LAA-_856_ = low-attenuation area of the lung with attenuation values below -856 Hounsfield units on full-expiration CT; R5-R20 = the difference from resistance at 5 Hz to resistance at 20 Hz; MMEF, %predicted = maximal mid-expiratory flow of percent predicted; FEF_50_, %predicted and FEF_75_, %predicted = forced expiratory flow at 50 and 75 of forced vital capacity of percent predicted.
